# HAZMAT Vehicle Reidentification in Road Tunnels Based on the Fusion of Appearance and Spatiotemporal Information

**DOI:** 10.1155/2023/3677387

**Published:** 2023-02-14

**Authors:** Lei Jia, Xiaobao Li, Wen Wang, Jianzhu Wang, Haomin Yu, Tianyuan Wang, Qingyong Li

**Affiliations:** ^1^Beijing Key Lab of Traffic Data Analysis and Mining, Beijing Jiaotong University, Beijing 100044, China; ^2^Frontiers Science Center for Smart High-Speed Railway System, Beijing Jiaotong University, Beijing 100044, China; ^3^Shenzhen Urban Transport Planning Center Co. Ltd., Shenzhen 518000, China

## Abstract

Vehicles transporting hazardous material (HAZMAT) pose a severe threat to highway safety, especially in road tunnels. Vehicle reidentification is essential for identifying and warning abnormal states of HAZMAT vehicles in road tunnels. However, there is still no public dataset for benchmarking this task. To this end, this work releases a real-world tunnel HAZMAT vehicle reidentification dataset, VisInt-THV-ReID, including 10,048 images with 865 HAZMAT vehicles and their spatiotemporal information. A method based on multimodal information fusion is proposed to realize vehicle reidentification by fusing vehicle appearance and spatiotemporal information. We design a spatiotemporal similarity determination method for vehicles based on the spatiotemporal law of vehicles in tunnels. Compared with other reidentification methods based on multimodal information fusion, i.e., PROVID, Visual + ST, and Siamese-CNN, experimental results show that our approach significantly improves the vehicle reidentification recognition precision.

## 1. Introduction

Hazardous materials (HAZMAT) could endanger the health and safety of people, environment, and property. With the increasing demand of HAZMAT, traffic accidents occurred frequently during HAZMAT transportation, and especially, a risk increase is generally observed in the presence of tunnels [1–3], which makes it of great importance to tighten regulation for vehicles transporting HAZMAT in tunnels.

HAZMAT vehicle reidentification (ReID) methods face the following challenges in tunnel scenes: (1) the strong reflection of the tank of a HAZMAT vehicle can cause large differences in its appearance under the uneven lighting conditions of a tunnel; (2) it is difficult to distinguish the HAZMAT vehicles with the same vehicle type effectively, due to their close appearance. However, there still remains a research gap both in HAZMAT vehicle data and in specialized algorithms. This motivates us to focus on the study of HAZMAT vehicle reidentification in tunnels.

**Figure 1:**
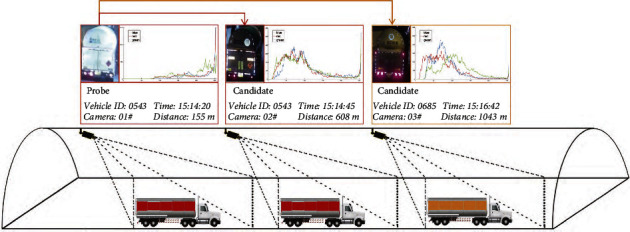
The HAZMAT vehicles are difficult to distinguish due to their close appearance. The reflection of the tank causes significant differences in its appearance under the variable lighting conditions in the tunnel.

Vehicle ReID aims to determine whether a vehicle image captured in nonoverlapping cameras belongs to the same vehicle in traffic monitoring scenarios. Existing methods mainly perform research on vehicle ReID based on the vehicle appearance [4]. However, due to the special and complex tunnel environment containing dim illumination and limited viewing field, it is more challenging for the tunnel vehicle ReID problem than that in open road scenes [5, 6]. Thus, large fluctuation can be seen by merely conducting tunnel vehicle ReID based on the appearance information. As shown in Figure 1, the red, green, and blue lines in each subfigure are RGB channel color histograms for each image. Vehicles for the second and third images may have similar appearance features, whereas they are actually two different IDs. From such instance, we can see that in real-world applications, it is extremely sensitive to environmental changes to merely perform vehicle ReID via appearance information.

To address the above problem, except for appearance information, the spatiotemporal information is further leveraged to improve vehicle ReID performance in recent works [7–9]. This is inspired by the fact that the vehicle movements follow some implicit motion pattern according to the traffic rules. However, due to the randomness of vehicle motion, it is difficult to accurately model the spatiotemporal motion laws of vehicles in the open road. But the traffic rules of vehicles in tunnels are more distinct than in the open road, such as vehicles are expected to move in one fixed direction within limited speed, and no U-turns. It leads to the urgent need for a special spatiotemporal model tailored to the tunnel scene.

Therefore, to realize HAZMAT vehicle ReID in tunnel scenes, this work proposes a vehicle ReID method based on the fusion of vehicle appearance and tunnel spatiotemporal information. For vehicle appearance modeling, a deep residual network (i.e., Resnet50 [10]) is chosen as a feature extractor to model the complex appearance variation of tunnel vehicle. Meanwhile, to capture the spatiotemporal cues between cameras and vehicles, we develop a novel spatiotemporal similarity metric to model the between-vehicle structure correlation as well as the camera-vehicle topological relationship.

Furthermore, the extracted appearance representation and the spatiotemporal model are combined to efficiently encode the appearance variation and movement pattern for the tunnel vehicles. Moreover, to evaluate the HAZMAT vehicle ReID problem in the tunnel scenes, we construct and release a real-world HAZMAT Vehicle ReID dataset, named by VisInt-THV-ReID, containing 10,048 images of 865 HAZMAT vehicles collected from four high-resolution cameras. These images were captured by 4 cameras in the tunnel. Each camera monitors a space with a range of 150 meters and takes around 3 pictures of vehicles with far, middle, and near distances, respectively. Each vehicle is attached by the camera mileage and the picture shooting time. According to the spatial coordinate transformation method [11], we infer the spatial positions of vehicles in tunnel from the perspective of camera monitoring and obtain their temporal information by comparing timestamps of monitoring cameras. We use the vehicle ReID to determine whether the HAZMAT vehicles are exiting the tunnel within a normal time. If one vehicle passes the tunnel more than once, we identify the HAZMAT vehicle with a different vehicle ID for each time in the dataset. More attention is paid to the driving condition of the HAZMAT vehicle each time when it passes through the tunnel. The proposed method is evaluated to be effective through exhaustive experiments on the VisInt-THV-ReID dataset.

The main contributions of this work are summarized as follows:

- We extend the scenarios of vehicle ReID task to the challenging problem of HAZMAT vehicle ReID in tunnel scenes and propose a method that fuses both appearance modeling and spatiotemporal mining for more precise vehicle ReID.
- We design a spatiotemporal metric approach based on the movement law of vehicles in road tunnels which brings in the description of between-vehicle structure correlation as well as the camera-vehicle topological relationship.
- We build a real-world tunnel HAZMAT vehicle ReID dataset, named as VisInt-THV-ReID. As far as we know, the released VisInt-THV-ReID is the first HAZMAT vehicle ReID dataset captured in the tunnel scenes, which is crucial for the promotion of automatic regulation of HAZMAT transportation. Exhaustive experiments demonstrate that the proposed method can generate a state-of-the-art performance.

The rest of this work is organized as follows: The review related works are presented in Section 2.  Section 3 details the proposed HAZMAT vehicle ReID method. In Section 4, we execute experiments for the evaluation of the proposed approach on VisInt-THV-ReID. Finally, we conclude this work in Section 5.

## 2. Related Work

Vehicle ReID in traffic monitoring scenarios can be seen as a part of multicamera tracking. Given an image of a vehicle in a specific area, the task is to find its image as captured under other cameras. This work studies vehicle ReID with spatiotemporal information fusion in tunnel scenes. We introduce related work from the aspects of vehicle ReID in tunnel scenes and multimodal information fusion.

### 2.1. Vehicle ReID Methods in Tunnels

Vehicle ReID in tunnel scenes is challenging due to low resolution, dim light, and dramatic changes in vehicle appearance. A vehicle is detected and tracked by each camera in road tunnels, and a detected vehicle is matched with the previous camera.

Frías-Velázquez et al. [6] proposed a probabilistic framework based on a two-step strategy that reidentifies vehicles in road tunnels. They built a Bayesian model that finds the optimal assignment between vehicles of connected groups based on descriptors such as trace transform signatures, lane changes, and motion discrepancies. Rios-Cabrera et al. [12] presented an integrated solution to detect, track, and identify vehicles in a tunnel surveillance application, taking into account practical constraints, such as real-time operation, imaging conditions, and decentralized architecture. AdaBoost [13] cascade is used for vehicle detection, and a comprehensive confidence score integrates the information of all stages of the cascade. Jelača et al. [14] proposed a real-time tracking method of multiple nonoverlapping cameras in a road tunnel monitoring scene, using AdaBoost for vehicle detection. The vehicle detector and a Kalman filter of average optical flow are used for tracking. The ReID algorithm applies the projection feature similarity of a radon transform between vehicle images. Chen et al. [15] proposed a spatiotemporal successive dynamic programming algorithm to identify vehicles between pairs of cameras. They extracted features based on Harris corner detection and OpponentSIFT descriptors, considering color information [16]. Zhu et al. [5] proposed a synergistically cascaded forest model to gradually construct the linking relationships between vehicle samples with increasing alternative random forest and extremely randomized forest layers.

The abovementioned methods generally focus on the extraction of hand-designed features of vehicle images, which can only show good performance in specific scenes. These manual features are susceptible to the interference of a complex tunnel environment, and they are difficult to improve the precision of ReID.

### 2.2. Methods Using Multimodal Information

As a vehicle is far from cameras and the illumination is insufficient, the image resolution is low. Due to their similarity, it is impractical to effectively identify HAZMAT vehicles without special markings only by appearance. Recent work on vehicle ReID has improved the model by combining multidimensional information of vehicle attributes such as type, color, time, and space information with appearance features.

To reidentify vehicles based on fusion different appearance information, Liu et al. [17] designed a network using BOW-SIFT [18], BOW-CN [19], and GoogLeNet [20] to extract texture, color, and semantic features, respectively. Handmade features are fused with the vehicle type and color features obtained through deep learning. Liu et al. [21] proposed PROVID, which makes full use of appearance features, license plates, camera locations, and semantic information to carry out a progressive search from coarse to fine in the feature domain and from near to far in physical space.

To reidentify vehicles based on spatiotemporal information, Zhong et al. [7] proposed a vehicle pose guide model using a spatiotemporal probability model based on the Gaussian distribution to predict the spatiotemporal motion of vehicles. A convolution neural network (CNN) was used to predict the driving direction of a vehicle and the results of visual appearance, and then, the driving direction and spatiotemporal models were fused. Shen et al. [8] proposed a two-stage framework incorporating complex spatiotemporal information to effectively regularize ReID results. A candidate visual-spatiotemporal path was generated by a chain Markov random field model with a deeply learned potential function. A Siamese-CNN + Path-LSTM model takes the candidate path and pairwise queries to generate a similarity score. Jiang et al. [9] proposed an approach with a multibranch architecture and a reranking strategy using the spatiotemporal relationship among vehicles from multiple cameras.

## 3. Method

### 3.1. Overview

Typically, a tunnel surveillance system consists of a series of cameras *C*={*C*_0_, *C*_1_, *C*_2_,…, *C*_*M*_}, with nonoverlapping visual receptive fields. $\overset{⟶}{A_{i}}$ denotes the 2048-dimensional appearance feature vector obtained from the *i*-th vehicle image through the image appearance feature extraction network, and $\overset{⟶}{S_{i}}$ denotes the spatiotemporal feature vector of the *i*-th vehicle collected by the camera. The spatiotemporal features involved are velocity *v*_*i*_, timestamp *t*_*i*_, and space position *l*_*i*_ of the tunnel.

We use *P*_*a*_(*i*, *j*) to represent the similarity of the appearance feature vectors of vehicles *i* and *j* from upstream and downstream cameras and *P*_*st*_(*i*, *j*) to represent the similarity of the spatiotemporal features of the vehicle pairs. *P*(*i*, *j*) is the probability that vehicle pairs are identical after fusing multimodal information. The inputs of the proposed model are vehicle image pairs (*i*, *j*) and their spatiotemporal features $\left(\overset{⟶}{S_{i}},\overset{⟶}{S_{j}}\right)$ involved velocity, timestamp, and space position in the tunnel. The output is the probability *P*(*i*, *j*) of whether the pair of vehicle images is the same vehicle.

**Figure 2:**
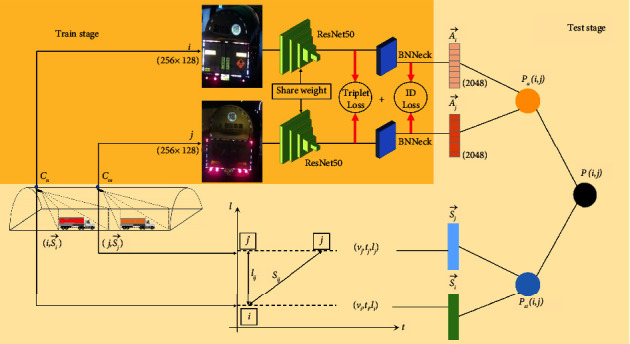
Vehicle ReID pipeline based on the fusion of appearance and spatiotemporal information.

The framework of the proposed method has three parts, as shown in Figure 2.

1. Similarity calculation of vehicle appearance features. Resnet50 [10] is used as the feature extractor to obtain a 2048-dimensional appearance feature vector of a vehicle.
2. Based on the spatiotemporal movement law of HAZMAT vehicles, we calculate the theoretical distance and the actual distance of the vehicle pairs. The tunnel spatial discrepancy *ε*_*ij*_ is used to evaluate the diversity between the theoretical distance and the actual distance.
3. Similarity calculation of multimodal information fusion. Based on parts 1 and 2, the spatiotemporal and appearance similarity of the input vehicle image pairs are summed with a weight. We rerank the vehicle similarity of fusion information.

### 3.2. Appearance Features of Vehicle ReID

**Figure 3:**
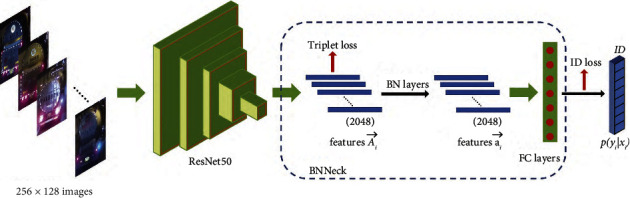
The framework of vehicle appearance modeling.

The vehicle appearance feature extraction network is shown in Figure 3. We use Resnet50 as the feature extraction backbone network and adjust each image to 256 × 128 pixels. Given an input image *x*_*i*_ with label *y*_*i*_, the predicted probability of *x*_*i*_ being recognized as class *y*_*i*_ is encoded with a softmax function, represented by *p*(*y*_*i*_|*x*_*i*_). ID prediction *p*(*y*_*i*_|*x*_*i*_) is used to calculate ID loss [22]. The model outputs ReID feature $\overset{⟶}{A_{i}}$ which is used to calculate triplet loss [23]. The output dimension of the full connection layer is changed to the number of vehicle IDs in the training dataset.

The ID loss treats the training process of vehicle ReID as an image classification problem [24], i.e., each identity is a distinct class. In the testing phase, the output of the pooling layer or embedding layer is adopted as the feature extractor. The identity loss is then computed by the cross-entropy.

(1) $$\begin{array}{c} L_{ID}=-\frac{1}{N}\overset{N}{\underset{i=1}{\sum }}\log\;\left(p\left(y_{i}\left|x_{i}\right.\right)\right)\text{,} \end{array}$$

where *N* represents the number of training samples within each batch.

The triple loss for feature extraction can reduce the intraclass distance of positive pairs and increase the interclass distance of negative pairs. Given a triplet (*x*^*a*^, *x*^*p*^, *x*^*n*^), including an anchor image *x*^*a*^, a positive *x*^*p*^, and negative *x*^*n*^, the triplet loss is formulated as follows:

(2) $$\begin{array}{c} L_{Tri}=\overset{N}{\underset{i=1}{\sum }}\left[{\left\|f\left(x_{i}^{a}\right)-f\left(x_{i}^{p}\right)\right\|}_{2}^{2}-{\left\|f\left(x_{i}^{a}\right)-f\left(x_{i}^{n}\right)\right\|}_{2}^{2}+\alpha \right]\text{,} \end{array}$$

where *α* is a margin and usually set to 0.3. *N* is the number of training samples within each batch. *f*(∙) stands for the appearance feature extractor.

In this work, we use ID loss and triplet loss together for optimizing the model. For image pairs in the embedding space, ID loss mainly optimizes the cosine distances while triplet loss focuses on the Euclidean distances. The feature vectors of the two losses are inconsistent in the embedding space. To address this problem, the BNNeck [22] is applied for more effective loss computation. BNNeck adds a batch normalization (BN) layer before the classifier FC layers of the model. The feature before the BN layer is denoted as $\overset{⟶}{A_{i}}$. We let $\overset{⟶}{A_{i}}$ pass through the BN layer to acquire a normalized feature $\overset{⟶}{a_{i}}$. In the training stage, the feature $\overset{⟶}{A_{i}}$ is used to compute the triplet loss. The feature $\overset{⟶}{a_{i}}$ is used to compute the ID loss. Finally, the triplet loss and ID loss are combined to optimize the model. To train the ReID model, we combine ID loss and triple loss as follows:

(3) $$\begin{array}{c} L=L_{ID}+L_{Tri}\text{.} \end{array}$$

In the test stage, the appearance features $\left(\overset{⟶}{A_{i}},\overset{⟶}{A_{j}}\right)$ for input image pairs (*i*, *j*) are generated using the vehicle appearance feature extraction network. We use the cosine distance to measure the similarity between features and is expressed as follows:

(4) $$\begin{array}{c} P_{a}\left(i,j\right)=\frac{\overset{⟶}{A_{i}}\cdot \overset{⟶}{A_{j}}}{\left\|\overset{⟶}{A_{i}}\right\|\left\|\overset{⟶}{A_{j}}\right\|}\text{.} \end{array}$$

### 3.3. Vehicle Spatiotemporal Features

The motion of the vehicle is limited by its speed and spatiotemporal motion. The time that the vehicle travels through a pair of cameras should be within a reasonable range. In a highway tunnel monitoring system, the driving speed of a vehicle is within the range of 10–80 km/h. The time interval of vehicle movement is affected by the camera installation position and the topological relationship of the tunnel and cameras. We analyze the motion law of the vehicle time interval between cameras in the VisInt-THV-ReID dataset. For each pair of cameras, the vehicle space interval can be modeled as a random variable that follows a certain distribution [6, 7].

**Figure 4:**
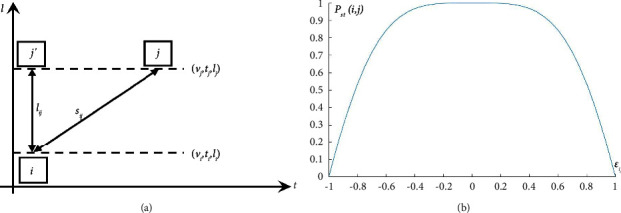
(a) Motion states of vehicles *i* and *j*. (b) Spatiotemporal similarity distribution in tunnels.

In order to derive the spatiotemporal similarity probability distribution of the vehicle, we propose a feature called spatial discrepancy. We introduce the spatial discrepancy by considering Figure 4(a). This figure shows the spatiotemporal graph that relates vehicle *i* observed in upstream camera with another vehicle *j* observed in downstream camera. The motion variables involved are velocity *v*_*i*_ of vehicle *i*, timestamp *t*_*i*_, and space position *l*_*i*_ of the tunnel. The state vector $\overset{⟶}{S_{i}}$ expresses the spatiotemporal state of vehicle *i*.

To construct the spatiotemporal similarity relationship between the vehicle pairs, we calculate the theoretical distance and the actual distance of the vehicle pairs and define the indicator *ε*_*ij*_ to calculate the diversity of the distances. According to the constant acceleration model, the theoretical distance of the vehicle is calculated as follows according to the upstream and downstream cameras of the tunnel:

(5) $$\begin{array}{c} s_{ij}=\frac{v_{i}+v_{j}}{2}\cdot \left(t_{j}-t_{i}\right)\text{.} \end{array}$$

The actual distance between the current position of the vehicle collected by the upstream and downstream cameras is expressed as follows:

(6) $$\begin{array}{c} l_{ij}=\left|l_{j}-l_{i}\right|\text{.} \end{array}$$

The spatial discrepancy *ε*_*ij*_ evaluates the fitness between the displacement estimate *s*_*ij*_ and the actual distance *l*_*ij*_ as stated in Figure 4(a). The tunnel spatial discrepancy is expressed as follows:

(7) $$\begin{array}{c} \varepsilon_{ij}=\frac{\left(s_{ij}-l_{ij}\right)}{\left|s_{ij}\right|+\left|l_{ij}\right|}\in \left(-1,1\right)\text{,} \end{array}$$

which is used to evaluate the diversity between the theoretical distance and the actual distance. The spatial discrepancy *ε*_*ij*_ is evaluated by the vehicle spatiotemporal features involving velocity, timestamp, and space position.

To maintain the consistency of the data structure of the multimodal data fusion, we maintain the consistency of the spatiotemporal similarity discriminant method with the appearance feature discriminant method and use the chord function to represent the spatiotemporal similarity probability distribution of the vehicle. The *P*_st_(*i*, *j*) is defined as follows:

(8) $$\begin{array}{c} P_{st}\left(i,j\right)=\cos\;\left(\varepsilon_{ij}^{2}\cdot \frac{\pi }{2}\right)\text{.} \end{array}$$

As shown in Figure 4(b), *P*_st_(*i*, *j*) increases as *ε*_*ij*_ tends to 0. Based on *P*_st_(*i*, *j*), we can determine candidate matching vehicles according to the spatiotemporal similarity in tunnels.

### 3.4. Vehicle ReID by Fusing Image and Tunnel Spatiotemporal Information

To make full use of the vehicle appearance and spatiotemporal information, we establish a multimodal information strategy. The vehicle ReID probability is defined as follows:

(9) $$\begin{array}{c} P\left(i,j\right)=\lambda P_{a}\left(i,j\right)+\left(1-\lambda \right)P_{st}\left(i,j\right)\text{,} \end{array}$$

where the weight coefficient, *λ* ∈ (0,1), is used to fuse the spatiotemporal and appearance similarity.

## 4. Experiments

### 4.1. VisInt-THV-ReID Dataset

**Figure 5:**
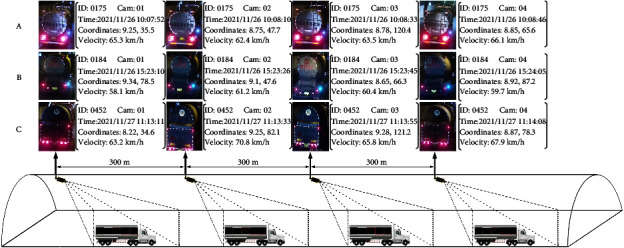
VisInt-THV-ReID dataset.

We verified the effectiveness of the proposed method on the VisInt-THV-ReID (The dataset is open-sourced at the following website: https://github.com/jialei-bjtu/VisInt-THV-ReID) dataset, which is collected from four cameras deployed in Taijia Expressway Linxian No. 3 tunnel in Shanxi province, China, providing high-definition video data of 6 million pixels and spaced at 300 meters. We collected video data for 10 hours daily over 3 days, from November 26 to 28, 2020, from 10:00 to 20:00. We annotated 10,048 pictures of 865 HAZMAT vehicles with their spatial position, speed, and timestamp information. To the best of our knowledge, this is the first open-source HAZMAT vehicle ReID dataset. The sample dataset is shown in Figure 5.

**Figure 6:**
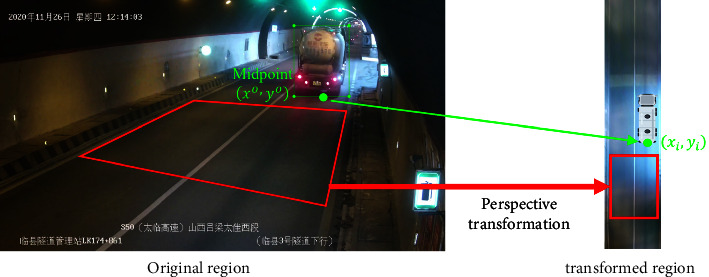
Coordinate transformation of vehicle position in tunnel space based on surveillance video.

To mark the spatiotemporal and speed information of a vehicle, we must transform its spatial coordinates. Perspective transformation is used to transform the vehicle driving area under the camera vision to a fixed-size rectangle [11], as shown in Figure 6.

The position (*x*_*i*_, *y*_*i*_) of a vehicle in the camera field of view in the tunnel is calculated as follows:

(10) $$\begin{array}{c} \left\{\begin{array}{c} \left[x^{\prime },y^{\prime },\omega^{\prime }\right]=\left[x^{o},y^{o},1\right]\cdot T\text{,}\\ T=\left[\begin{array}{ccc} a_{11} & a_{12} & a_{13}\\ a_{21} & a_{22} & a_{23}\\ a_{31} & a_{32} & a_{33} \end{array}\right]\text{,}\\ \left[x_{i},y_{i}\right]=\left[\frac{x^{\prime }}{\omega^{\prime }},\frac{y^{\prime }}{\omega^{\prime }}\right]\text{,} \end{array}\right. \end{array}$$

where *x*_*i*_ is the lateral distance of the vehicle from the left wall of the tunnel, *y*_*i*_ is its longitudinal distance from the current camera installation position, (*x*^*o*^, *y*^*o*^) is the lower midpoint of the vehicle object detection box in the image, and *T* is the transformation matrix defining the mapping between the original region and the transformation region. Using the image sequence taken by the surveillance camera, the speed of vehicle *i* in the tunnel can be obtained as follows:

(11) $$\begin{array}{c} v_{i}=\left(\sqrt{x_{i}^{2}+y_{i}^{2}}-\sqrt{x_{i-1}^{2}+y_{i-1}^{2}}\right)\cdot f\text{,} \end{array}$$

where *f* is the frame rate of the monitoring camera, the spatial position vector *l*_*i*_ obtained by the camera at time *t*_*i*_ is (*x*_*i*_, *y*_*i*_), and the spatiotemporal vector of vehicle *i* is $\overset{⟶}{S_{i}}\left(v_{i},t_{i},l_{i}\right)$.

We trained and tested the model on the VisInt-THV-ReID dataset, whose 10,048 images of 865 HAZMAT vehicles were divided into training, query, and test sets at a 10 : 1 : 9 ratio. The training set had 433 HAZMAT vehicles and 4980 images. There were 432 HAZMAT vehicles in the query and test sets, with 432 vehicle images in the query set and 4636 in the test set.

### 4.2. Experimental Settings

The mAP [21] and cumulative matching characteristic (CMC) curve [25] were used to evaluate the performance of the proposed method on the VisInt-THV-ReID dataset. The average precision for a query image is calculated as follows:

(12) $$\begin{array}{c} AP=\frac{\sum_{k=1}^{n}P\left(k\right)\cdot gt\left(k\right)}{N_{gt}}\text{,} \end{array}$$

where *n* is the number of images in the test set, *N*_gt_ is the number of ground truths, *P*(*k*) is the current precision result of the *k*-th query image, and gt(*k*) is an indicator function. When the matching result of the *k*-th query image is correct, gt(*k*)=1, and gt(*k*)=0 when it is incorrect.

The mAP is calculated as follows:

(13) $$\begin{array}{c} mAP=\frac{\sum_{q=1}^{Q}AP\left(q\right)}{Q}\text{,} \end{array}$$

where *Q* is the number of pictures in the query dataset. The CMC curve shows the probability that the correct matching image of the vehicle appears in the candidate lists. The CMC of the *k*-th position is as follows:

(14) $$\begin{array}{c} CMC\left(k\right)=\frac{\sum_{q=1}^{Q}gt\left(q,k\right)}{Q}\text{,} \end{array}$$

where gt(*q*, *k*) is an indicator function, which equals 1 when the ground truth of the *q* query image appears before the *k* position. We also used Rank-1, Rank-5, Rank-10, and Rank-20 in the field of ReID to evaluate the model.

### 4.3. Ablation Study

Table 1 compares the experimental results of the multimodal fusion ReID method with those of Visual and ST-COS, which are appearance-based and spatiotemporal-based, respectively.

The method of Visual achieved 89.7% mAP and 96.3% Rank-1. The method of ST-COS achieved 85.5% mAP and 71.3% Rank-1. The fusion method Visual + ST-COS achieved 99.7% mAP and 99.8% Rank-1. The mAPmAP of the fusion method increases by 142% and 10% compared to Visual and ST-COS and the Rank-1 rises by 3.5% and 28.5%.

The above results show that the multimodal information fusion method is superior to the use of appearance or spatiotemporal information alone and verify the effectiveness of the proposed multimodal information fusion method.

### 4.4. Comparison with Baselines

Table 2 shows the recognition precision of three baseline methods, PROVID [21], Visual + ST [7], and Siamese-CNN [8], comparing to that of Visual + ST-COS on the VisInt-THV-ReID dataset.

#### 4.4.1. Appearance Feature Extraction and STR Spatiotemporal Fusion (PROVID)

The method of PROVID extracts the appearance features of HAZMAT vehicles by the Resnet50 network and uses the STR method to measure the spatiotemporal relationship [21]. The STR is defined as follows:

(15) $$\begin{array}{c} STR\left(i,j\right)=\frac{T_{i}-T_{j}}{T_{\max}}\cdot \frac{\delta \left(C_{i},C_{j}\right)}{D_{\max}}\text{,} \end{array}$$

where *T*_*i*_ and *T*_*j*_ are the timestamps for the vehicles *i* and *j* captured by the cameras. *T*_max_ is the maximum time interval of vehicles passing through the tunnel. *δ*(*C*_*i*_, *C*_*j*_) is the actual distance between the current position of the vehicles collected by the upstream and downstream cameras, and *D*_max_ is the global maximum distance between any vehicles. We set *D*_max_ as the length of the tunnel.

#### 4.4.2. Visual + ST

The method of Visual + ST extracts the appearance features of HAZMAT vehicles with the Resnet50 network and uses a spatiotemporal model based on the Gaussian distribution to predict the probability of vehicles [7]. *P*_stG_(*i*, *j*) presents the similarity of the spatiotemporal features of vehicle pairs, and it is defined as follows:

(16) $$\begin{array}{c} P_{stG}\left(i,j\right)=e^{\left(-10\cdot \varepsilon_{ij}^{2}\right)}\text{,} \end{array}$$

where *ε*_*ij*_ is the tunnel spatial discrepancy as defined in equation (7).

#### 4.4.3. Siamese-CNN

The method of Siamese-CNN uses a Resnet50 network to extract the appearance features of HAZMAT vehicles, and a multilayer perception network is applied to obtain their spatial and temporal relationships [8]. The spatiotemporal branch computes the spatiotemporal compatibility. Given the timestamps (*t*^*i*^, *t*^*j*^) and the positions (*l*^*i*^, *l*^*j*^) of vehilces, the input features of the branch are calculated as their time difference ∆t(*t*^*i*^, *t*^*j*^) and spatial difference ∆d(*l*^*i*^, *l*^*j*^). The scalar spatiotemporal compatibility is obtained by feeding the concatenated features, [∆*t*(*t*^*i*^, *t*^*j*^), ∆*d*(*l*^*i*^, *l*^*j*^)]^*T*^, into a multilayer perception with two fully connected layers. The outputs of the two branches are concatenated and input into a 2 × 1 fully connected layer with a sigmoid function to obtain the final compatibility between the two states. Siamese-CNN takes all visual, spatial, and temporal information into consideration.

**Figure 7:**
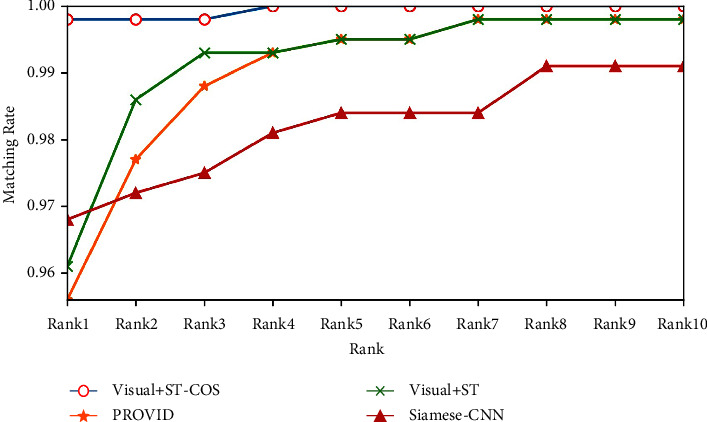
CMC curves on VisInt-THV-ReID dataset.

The results show that the proposed method achieves the best performance. It improves *mAP* and Rank-1 by 9.7% and 4.2%, respectively, compared with PROVID. This indicates that the STR spatiotemporal measurement method is not accurate enough to express the spatiotemporal information of vehicles in road tunnels. Compared with Siamese-CNN, the proposed method improves mAP and Rank-1 by 17.5% and 3.0%. Since Siamese-CNN uses a multilayer perception network to train the spatial and temporal information of vehicles, the difficulty of model training is decreased and the precision is not ideal. Compared with Visual + ST, the proposed method improves mAP and Rank-1 by 8.9% and 3.7%, respectively. This shows that the proposed cosine spatiotemporal model can more accurately express the spatiotemporal state of a tunnel compared with Gaussian distribution. The CMC curves of all methods are shown in Figure 7.

### 4.5. Parameter Analysis

We experimented with the parameters of *λ* in the interval of 0.1–0.9. The best fusion result is achieved when *λ* equals 0.35. The comparison results of the parametric experiments are shown in Table 3. It can be observed from the table that a larger *λ* would cause appearance features to dominate vehicle identification, while a smaller *λ* causes spatiotemporal information to dominate. Table 3 shows that *λ* can have an important effect on the fusion results, and *λ* is relatively insensitive to the results in the interval 0.3–0.7.

## 5. Conclusion and Future Work

In this study, we presented a vehicle ReID method based on the fusion of vehicle appearance and tunnel spatiotemporal information for the task of HAZMAT vehicle ReID in road tunnels. The proposed method was evaluated on the VisInt-THV-ReID dataset. This study could play a role in promoting HAZMAT vehicle monitoring and traffic safety management in road tunnels.

Our future work has two aspects. Based on vehicle ReID research, we will study multicamera vehicle tracking technology to collect vehicle trajectories. In addition, we will use the time-to-collision (TTC) to indirectly evaluate safety and study a tunnel accident risk prediction model based on the traffic flow state.

## Figures and Tables

**Table 1:** Results of ablation experiment.

Methods	mAP (%)	Rank-1 (%)	Rank-5 (%)	Rank-10 (%)	Rank-20 (%)
Visual	89.7	96.3	99.5	99.5	99.8
ST-COS	85.5	71.3	85.9	98.8	100
Visual + ST-COS	99.7	**99.8**	**100**	**100**	**100**

The bold values in Table 1 are the best values from the same column of data.

**Table 2:** Results of comparative experiments.

Methods	mAP (%)	Rank-1 (%)	Rank-5 (%)	Rank-10 (%)	Rank-20 (%)
PROVID	90.0	95.6	99.5	99.8	99.8
Visual + ST	90.8	96.1	99.5	99.8	99.8
Siamese-CNN	82.2	96.8	98.4	99.1	99.3
Our method	**99.7**	**99.8**	**100**	**100**	**100**

The bold values in Table 2 are the best values from the same column of data.

**Table 3:** Experimental results of coefficient *λ* under different values.

Results	*λ* = 0.1	*λ* = 0.2	*λ* = 0.3	*λ* = **0.35**	*λ* = 0.4	*λ* = 0.5	*λ* = 0.6	*λ* = 0.7	*λ* = 0.8	*λ* = 0.9
mAP	88.2	97.3	99.3	**99.7**	99.7	99.7	99.7	99.5	98.6	96.2
Rank-1	81.0	98.8	99.8	**99.8**	99.8	99.7	99.8	99.8	99.5	99.1
Rank-5	94.7	100	100	**100**	99.8	99.8	99.8	99.8	99.8	99.8
Rank-10	99.8	100	100	**100**	100	100	99.8	99.8	99.8	99.8
Rank-20	100	100	100	**100**	100	100	100	99.8	99.8	99.8

The bold values in Table 3 are the best values from the same column of data.

## Data Availability

The data that support the findings of this study are openly available in GitHub at https://github.com/jialei-bjtu/VisInt-THV-ReID.
